# Concealed Wireless Warning Sensor Based on Triboelectrification and Human-Plant Interactive Induction

**DOI:** 10.34133/2021/9870936

**Published:** 2021-04-29

**Authors:** Yange Feng, Enrico Benassi, Liqiang Zhang, Xiaojuan Li, Daoai Wang, Feng Zhou, Weimin Liu

**Affiliations:** ^1^State Key Laboratory of Solid Lubrication, Lanzhou Institute of Chemical Physics, Chinese Academy of Sciences, Lanzhou 730000, China; ^2^Qingdao Center of Resource Chemistry and New Materials, Qingdao 266100, China; ^3^Public Technical Service Center, Lanzhou Institute of Chemical Physics, Chinese Academy of Sciences, Lanzhou 730000, China; ^4^Novosibirsk State University, 1 Pirogova Str., Novosibirsk 630090, Russia

## Abstract

With the continuous development of artificial intelligence, the demand for sensors with simple preparation and strong concealment continues to increase. However, most of the high-sensitivity sensors have complex manufacturing methods, high costs, and single functions. In this paper, a sensitive motion sensor based on the triboelectric interaction between a living plant and the human body was designed to detect the real-time movements of human beings and provide danger warning. A certain relationship exists between the triboelectric signal and the distance between the plant and the human body, with effective signals being detected in the range of 1.8 m. In addition, the triboelectric signal generated by each person is unique like a fingerprint, which can be used for biometrics. On the basis of the triboelectric signal, a wireless character entry warning system is designed. This sensor can not only send out a wireless warning signal at a specific distance but also allow one to receive the warning information synchronously on a mobile phone in real time. The wireless movement sensor receives signals through a living plant, and it has the characteristics of convenient use, strong concealment, and shielding difficulty. This sensor has the potential to be widely used in person recognition, danger warning, and motion monitoring.

## 1. Introduction

A sensor is used to detect physical signals and transform the detected signals into electrical signals or other forms of outputs according to a specific rule. With the aid of sensors, many important physical properties can be accurately detected, such as temperature [1, 2], pressure [3, 4], light intensity, and magnetic field strength [5, 6]. To detect the information, a variety of sensors have been designed based on different principles, such as strain, resistance, piezoelectricity, and triboelectricity. [7–9] Although these sensors can effectively detect human movement, they have a relatively complex structure and poor concealment. In some special fields, sensors with the characteristics of simple preparation, strong concealment, self-powered, and wireless transmission, are still urgently needed. As a ubiquitous physical phenomenon, triboelectric signals can be generated during a contact or friction process, which is mainly due to the electron-transfer ability between two frictional materials [10–12]. Some studies have proven that bio-based materials such as leaves and celluloses can be used to fabricate triboelectricity to power electronic devices [13–16]. However, these biological materials have all been further processed to a certain extent so that they can be prepared into the structure of a traditional triboelectric nanogenerator, instead of experiments being conducted directly on living plants. A potentially interesting and important method is to design power-generating units or sensing units based on living plants that exist widely in nature. If this kind of plant-based sensor can be realized, then a sky-eye system with plants in the earth as the terminal sensor could be established to provide real-time danger warning.

The phenomena of frictional electrification and contact electrification are commonly observed in daily life experience [17, 18]. Thus, increasing research interest has been focused on triboelectricity and its applications in various areas [19–21], which provides a potential way for designing a universally triggerable sensor based on triboelectrification and electrostatic induction. As a special type of electricity energy, triboelectricity can also be utilized through a triboelectric nanogenerator for some self-powered devices, as first reported by Wang et al. in 2012 [22]. The output performances of a triboelectric nanogenerator are affected by many environmental factors, such as humidity, temperature, pressure, and gas concentration. Thus, triboelectricity has been widely used as a detectable signal in sensor devices to monitor changes in the environment [8, 23–26]. The detection of triboelectric signals also sheds light on a possible way of perception and communication between living beings and the surrounding nature [27–29]. For example, the location of a moving body could be monitored by detecting and tracking the triboelectric signals produced by its movement [30–32]. With the continuous development of artificial intelligence, the demand for sensors with simple preparation and strong concealment continues to increase. However, most of the highly sensitive sensors have complex manufacturing methods, high costs, and single functions. Through the collection and processing of the triboelectric and induced electrical signals between humans and plants to realize the interaction relationship between humans and plants, a new way of designing concealed wireless warning sensors is provided.

In this work, a movement sensor based on the triboelectric interaction between a living plant and the human body was designed to detect the real-time movements of human beings. When a person moves near a plant connected to a detector, a regular AC signal can be monitored in real time. The whole system, except for the detector, is constituted of natural materials. On the basis of the triboelectric signal, a wireless character entry warning system is designed. When people step close to a plant, the triboelectric electricity will be transmitted to the signal transmitter of the wireless sensor and will trigger an early warning signal. Then, the acceptor receives the alert signals and sends out an alarm. Meanwhile, the alert information will be uploaded to an online server and can be received in real time by a mobile phone. The wireless movement sensor receives signals through a living plant, making it a promising device for application in the field of movement detection. As shown hereinafter, the detected signal is characteristic of each individual moving body and the sensor might therefore be utilized for identity recognition or approach alarm.

## 2. Results

**Figure 1:**
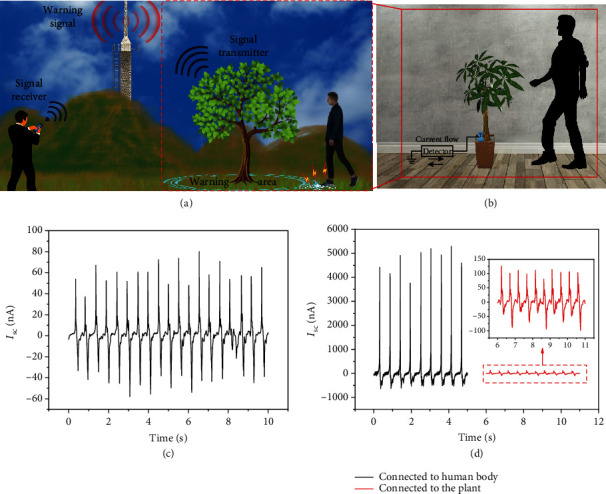
(a) Schematic of the green plant-based danger warning system. (b) Schematic image and photograph of the operating process of the motion sensor part. (c) Detected short-circuit current during the operating process. (d) *I*_sc_ comparison under different wiring methods.

The scheme of the green plant-based wireless danger monitoring system is depicted in Figure 1(a). The most critical part of this system is the motion sensor on the right part of Figure 1(a). To detect the sensor signal, one port of the detector is directly connected to the plant through lead and a Ti sheet inserted into the soil, whereas the other port is connected to the ground. The device can be divided into two modules, namely, the charge generator and the signal receptor. The frictional material (shoes) and the living plant function as the signal generator and receptor, respectively. The photo of the operating process of the movement sensor system is shown in Figure 1(b). When a person steps in front of the plant, a regular alternative current of 80 nA is revealed by the detector (Figure 1(c)). We conducted another test to prove that the tree functions more like a wireless receiver. As shown in Figure 1(d), when the human body is directly connected to the detector, the detected current is more than 50 times greater than when it is connected to a plant. This condition is more similar to signal attenuation in wireless transmission than in wired transmission. When *Pachira macrocarpa* is replaced with *Epipremnum aureum*, the maximum value of the current signal has changed, but the overall peak shape still has many similarities, as shown in Figure S1a. In other words, other plants can also be used as alternative options to realize the sensor warning. The variety of plants in nature also makes this early warning sensor system more concealed and practical. The value of the detected current intensity depends on several factors, some of which we shall investigate hereafter.

**Figure 2:**
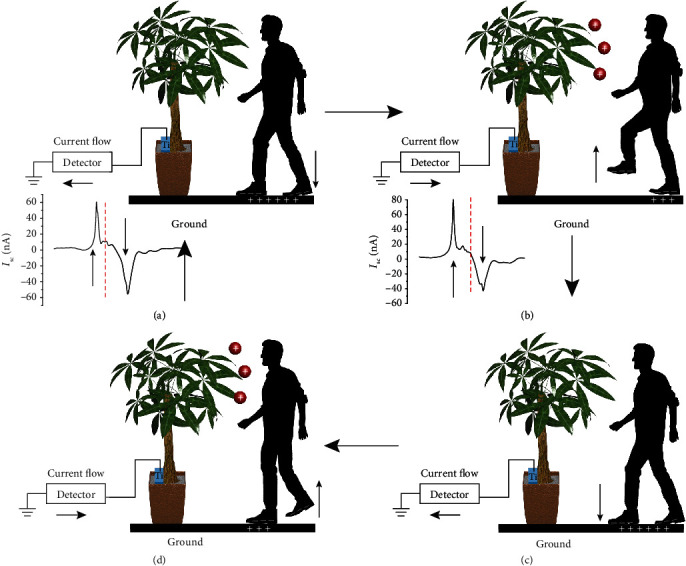
Working mechanism of the green plant-based movement sensor: (a) right foot stepping down on the ground, (b) left foot stepping up, (c) left foot stepping down, and (d) right foot stepping up.

Figure 2 depicts a probable schematic of the working mechanism of the wireless movement sensor. When people step on the ground, triboelectricity will be generated on the ground and on the shoe's sole with opposite charges (Figure 2(a)). After the shoe's sole steps up and becomes separated from the ground, a difference in electric potential Δ*V* and an electric field will be generated between the shoe's sole and the surface of the ground, which drives the electrons to flow through the external circuit (Figure 2(b)). When the shoe's sole approaches back to the ground, to balance the potential, the electrons flow from the ground to the detector, thus generating an opposite current signal (Figure 2(c)). After the shoe's sole steps up and becomes separated from the ground surface again, a difference in electric potential Δ*V* and an electric field will be generated between the shoe's sole and the ground surface, which drives the electrons to flow through the external circuit (Figure 2(d)). When the shoe's sole steps up and down, a regular AC current can be detected by the current amplifier.

**Figure 3:**
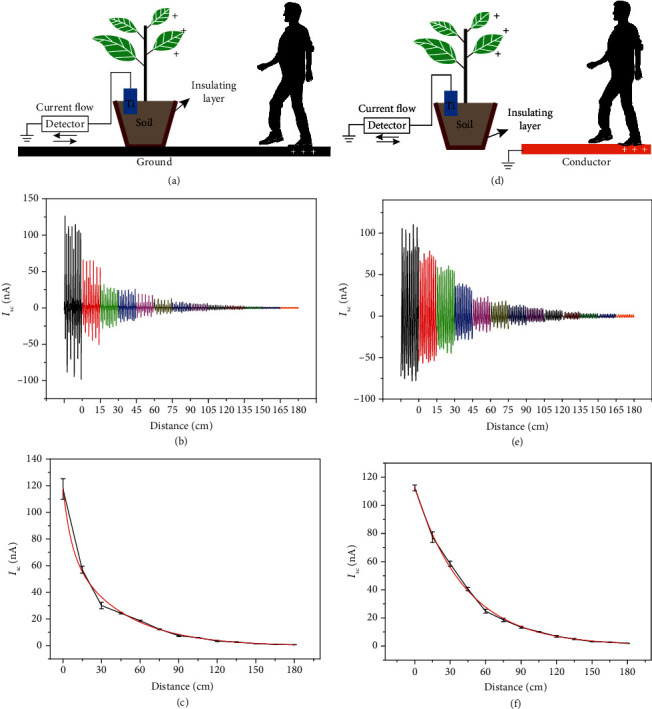
Detected signals at different distances, considering steps on the ground (a–c) and steps on a conductor (d–f).

The sensor is designed to detect real-time movements. Thus, the relationship between distance and current is an essential point to determine. With focus on this scope, several experiments were conducted on different floor types, including ground and grounded copper foil floor (Figures 3(a) and 3(b)). The test results in Figures 3(b) and 3(e) show that whether on the ground or on a grounded copper plate, the detected current value decreases as the distance increases. However, judging from the relationship curves between current and distance in Figures 3(e) and 3(f), the results between the fitted curve and the actual curve when a person steps on a grounded copper plate are more consistent. For the aforementioned considerations, concerning the mechanism of transmission of the signal, the trend in both cases is expected to vary as a decreasing function of the distance such as for wave attenuation, *viz*.:

(1) $$\begin{array}{c} I_{\text{SC}}\left(r\right)=\frac{A}{4\pi {\left(r-r_{0}\right)}^{2}}, \end{array}$$

where *A* indicates a constant that depends on the materials used in the experiment. Equation (1) is a reasonable approximation for short to medium distances from the receiver. The values of *A* upon regression of the experimental data are 1.90 × 10^−7^ A · m^2^ (for copper; adj − *R*^2^ = 0.962) and 0.86 × 10^−7^ A · m^2^ (for ground; adj − *R*^2^ = 0.942). We can conclude that the grounded conductor is more feasible than the ground floor as the triboelectric partner with the human body, showing a larger sensitivity. We have proceeded an experiment to figure out the resolution of the motion sensor. As shown in Figure S2, when the test distance interval is 2 cm, the output at different distances can be clearly distinguished. When the distance interval is less than 2 cm, the output cannot be clearly distinguished. Therefore, the resolution of this motion sensor is 2 cm.

We also tested the sensitivity of this sensor under different conditions. We tested the strength of the sensor signal under four conditions: shoes, barefoot, socks, and aluminium foil-covered soles. The strongest sensor signal is obtained when the person is wearing shoes, while the weakest signal is obtained when aluminium foil covers the sole, according to Figure S3a. This condition occurred mainly because the sole is a triboelectric negative material, which generates more triboelectric charge when it comes in contact with the ground. The electron-transfer ability between the aluminium foil and the ground is very close, which is why the generated frictional charge is small. Therefore, when the aluminium foil is in contact with the ground, little triboelectricity is generated. In other words, polymer materials that are easily negatively charged when rubbed against the ground have higher sensing sensitivity. To explore how the humidity of the soles affect the detected signal, a detailed experiment was carried out and relative results are shown in Figure S3b. When shoe soles are dry, the current value is normal. When the sole of the shoe gets wet, the current value obtained by the test becomes smaller. However, as the testing process continues, the water in the soles gradually evaporates, and the current obtained by the test gradually returns to the normal value. At the same time, we sprinkled more water on the ground to keep the whole contact process in a completely wet state. The results obtained are similar to the initial results when the soles are wet. In other words, the degree of wetness of the sole will indeed affect the current value obtained by the test. Compared with the shoe sole under dry conditions, the result obtained with a completely wet shoe sole shows that the detected current value is attenuated by more than half. Furthermore, we have carried out a detailed experiment to explore whether swinging the arm would affect the results. As shown in Figure S3c, when swinging the arm, the detected signal is much smaller than the signal generated when stepping. In other words, the process of swinging the arm can hardly affect the detected signal.

**Figure 4:**
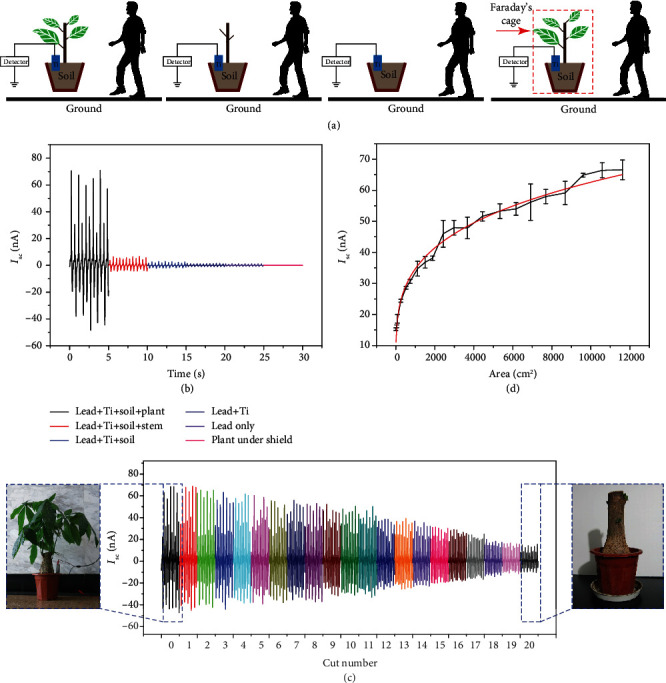
(a) Schematic representations of different types of experiments performed and (b) the corresponding detected current profiles. (c) Detected current as a function of the number of leaves that remained on the plant. (d) Relationship between the detected current and total surface area of leaves that remained on the plant.

In addition to the distance and material, the plant itself and the factors that can affect the plant may have a certain impact on the detection signal of the entire sensor system. Further parameters were tested to optimize the efficiency of the device (Figure 4). First, six different groups of experiments were designed and performed to determine whether the plant is indeed an essential element for this new type of sensor. Figure 4(a) provides a schematic representation of four primary groups of experiments, namely, the usage of a whole plant, the usage of a plant with the stem only (without leaves), a system without the plant, and a system where the plant is shielded by a Faraday cage. As shown in Figure 4(b), the values of the current detected in the case of the whole plant without a shield are the largest, whereas current detected in the case of the shielded system is the least among the six groups. Hence, we can conclude that the plant is an essential component of the system. When the leaves of the plant were progressively removed until only the stem remained (Figure 4(c)), the current output showed a significant decrease, which should be correlated with the value of the surface area of leaves working as antennae catching the electric signals (Figure 4(d)). The measured values of the current were fitted using

(2) $$\begin{array}{c} I_{\text{SC}}\left(a\right)=I_{0}+I_{\max}\left(1-e^{-a/a_{0}}\right), \end{array}$$

where *a* indicates the total surface area of the remaining leaves, *I*_0_ is the residual current intensity (when no leaves are left but only the stem remains), *I*_max_ is the maximum current intensity that can be captured with a hypothetical plant having an infinite number of leaves, and *a*_0_ is the characteristic area, i.e., a parameter indicating the effectiveness of the type of leaves on the detection process. The parameters obtained upon nonlinear fitting are *I*_0_ = (18.9 ± 1.3) nA, *I*_max_ = (45.6 ± 2.0) nA, and *a*_0_ = (0.33 ± 0.4) m^2^ (adj − *R*^2^ = 0.971). The results show the relevance of the role played by the leaves in the system's efficiency in terms of amount and intrinsic characteristics. When the stem, soil, and the Ti sheet were removed in that order, the detected current progressively decreased. The plant in its entirety, therefore, plays a leading role in the sensitivity of the device. Meanwhile, three plants were prepared with similar overall sizes, and corresponding comparative experiments were carried out. It can be seen from Figure S4 that these three plants have similar sizes. From the results, *Asparagus* ferns obtained the largest current during the test, followed by *Pachira macrocarpa* and *Osmanthus fragrans*. On the whole, the maximum current values obtained by the three plants are relatively close. From the side, it can also prove our research results on plant area and detected current output.

When the material of the shoe's sole and the ground surface is a polarizable dielectric (e.g., silicon rubber), the charges generated by the triboelectric effect will remain localized in their position; we should consider that the shoe's sole does not entirely contact with the ground surface due to the specific shape and pattern manufactured. Therefore, only a fraction of the surface area will carry charges. If the pressure on the shoe's soil is enhanced (e.g., when some load is carried or when stepping occurs more rapidly and frequently), then the amount of charge generated will also increase, which can be proved by Figure S5 in the Supporting Information. However, increasing foot pressure and body weight is not a standard approach. Therefore, a relatively standard test method needs to be designed to characterize the influence of the area of the friction material and the applied pressure on the triboelectric performance.

**Figure 5:**
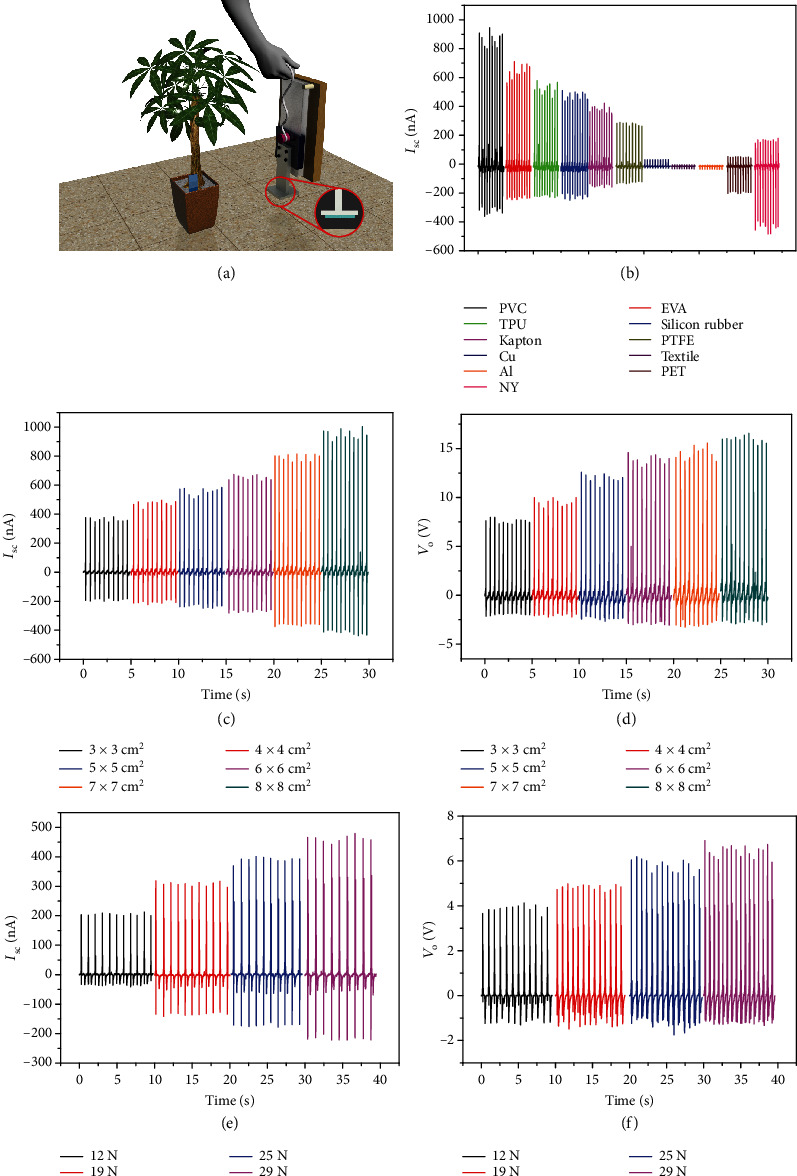
(a) Schematic image of the standard test model for quantitatively characterizing the influence of pressure and area of friction materials on triboelectric signals; (b) triboelectric signals generated by different materials; (c, d) current and voltage output generated by silicon rubber with different sizes; (e, f) current and voltage output generated by silicon rubber under different contact pressure levels.

**Figure 6:**
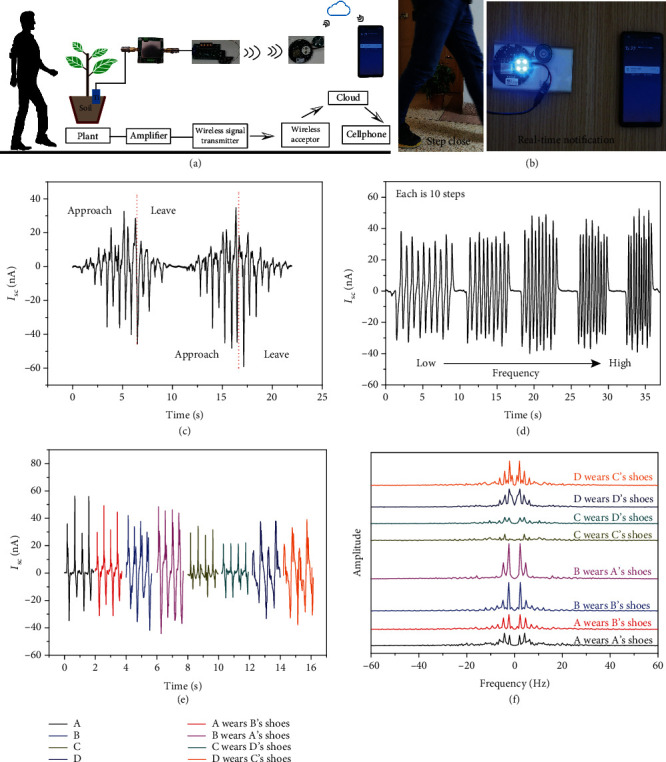
(a) Scheme of the green plant-based monitoring sensor; (b) photograph of the green plant-based monitoring sensor in practical use; (c) detected signals when a human body steps close to and away from the plant; (d) detected signals under different step frequencies; (e) detected signals for four different individuals wearing different shoes; and (f) FFT analysis of four different individuals wearing different shoes.

Figure 5(a) shows the device used to simulate human stepping, while the inset is an enlarged view of the impact part. Different materials are pasted on the front of the impact rod, and the contact pressure is controlled by the drop height of the impact rod. Concerning the signal generator, the chemical nature of the frictional material affects the intensity and wave shape of the signal. PTFE and nylon were chosen as the frictional materials to replace the shoes, because PTFE approximates negative triboelectric polarity, while nylon approximates positive triboelectric polarity, according to previous studies [11, 33]. The value of the triboelectric polarity of the ground (mainly composed of SiO_2_) is between the values of PTFE and nylon. Therefore, the generated signals have opposite polarity when PTFE or nylon contacts with the ground (Figure S6a). This finding verifies the authenticity of the signal detected by an ammeter. Although the produced signals are opposite, an interesting detail is that the maximum value produced by PTFE is smaller than that of NY, which is not exactly the same as the fact that PTFE can produce more triboelectric charge under normal circumstances. Therefore, testing the output performance of the plant-based triboelectric system when different materials are in contact with the ground is meaningful for the practical application of this triboelectric system. As can be seen in Figure 6(b), when different materials contact with the ground, the triboelectricity generated is different. PVC, as a triboelectric negative material, generates the highest current output when it comes in contact with the ground; nylon, as a triboelectric positive material, also generates a relatively high output when it comes in contact with the ground. Obviously, this test result will be helpful for choosing the right friction layer material. The experimental results are consistent with those predicted theoretically by means of the model derived in the Supporting Information, *viz.* 85.6 nA for PTFE and 50.1 nA for NY put in contact with a silica interface (the theory slightly overestimates the absolute values of current intensity with respect to the experiments, due to the ideality of the systems simulated, but the ratio between the two values is consistent with the one revealed experimentally). The contact area between the material and the ground will also affect the current signal detected by the triboelectric sensing system. To verify this result, we chose silicone rubber as the friction layer material to contact the ground because many shoe soles have silicone rubber components and silicone rubber is relatively soft. Thus, the contact area when in contact with the ground is closer to the contact area in the ideal state. As shown in Figures 5(c) and 5(d), the detected current and voltage values increase with the increase in the contact area. The results in Figure 5(e) also show that a linear relationship exists between the contact area and the current value (Figure S6b). Similarly, a relationship exists between the detected current and voltage values and the applied pressure. Here, we control the pressure during contact by controlling the height at which the impact rod falls. As the contact pressure increases from 12 N to 29 N, the current and voltage values detected by the triboelectric sensing system also gradually increase, as shown in Figures 5(e) and 5(f). Figure S6c also shows that an obvious linear relationship exists between the contact pressure and the short-circuit current.

The movement sensor was shown to be sensitive to certain specific circumstances. The current intensity gradually increases as the person approaches the plant, reaches a maximum value in correspondence with the plant, and gradually decreases as the person steps away from the plant (Figure 6(c)). This finding can be explained in a straightforward manner by using the previous results (Figure 3 and equation (1)). This result is important for danger warning. Therefore, we designed a wireless warning device based on this sensing signal, which is shown in Figures 6(a) and 6(b) and the supplemental Video S1. When the human body approaches the tree and reaches a certain range, the transmitter will emit wireless signals. At the same time, the wireless receiver will receive the corresponding signals and issue an alarm, and the mobile phone will receive the alarm signal in real time. The frequency of the detection signal can also be used to calculate the number of steps according to the result in Figure 6(d) and Video S2. As shown in the figure, although the stepping frequency is different, the number of steps can be calculated by detecting the number of peaks.

The most outstanding result of this work is the discovery that the shape of the detected current signals has peculiar features that are dependent on the walking individual (Figure 6(e)), hence possibly representing a sort of “fingerprint” for identity recognition. The signals are almost unchanged in shape when people wear different shoes. To provide quantitative information about the detected differences, the current signal was subjected to FFT analysis. The amplitudes of the electric signals (Figure 6(f)) show harmonics that are characteristic of the individual, as it happens for voice timbre. This result makes the proposed device applicable in many situations, including identity recognition and home security.

## 3. Discussion

In this paper, a movement sensor based on the triboelectric interaction between a living plant and the human body is designed to detect real-time movements. This paper presents the first case where this triboelectric type of interaction between humans and plants is described. On the basis of the triboelectric signal, a wireless character entry warning system is designed. When people step close to the plant within 1.8 m, the triboelectric electricity is transmitted to the signal transmitter of the wireless sensor and triggers an early warning signal. Meanwhile, the alert information is uploaded to an online server, and a mobile phone can receive the alert information in real time. The wireless movement sensor receives signals through a living plant, which has a potential application in the field of movement detection. Therefore, the detected signal is characteristic of the body movements of each individual and the sensor might therefore be utilized for people recognition or danger warning.

## 4. Materials and Methods

### 4.1. Materials

A green plant (*Pachira macrocarpa*) was purchased from a local market. The Ti sheet (99.99%) was purchased from ZhongNuo Advanced Material (Beijing) Technology Co., Ltd., China. The online real-time notification system is supported by the MiJia App (Xiaomi Technology Co., Ltd., China). The wireless signal transmitter and wireless signal receiver are modified by Linptech smart doorbell (Wuhan Linptech Co., Ltd., China).

### 4.2. Fabrication of the Movement Sensor

A Ti sheet is inserted into the soil of the green plant with a copper lead connected to the current detector, whereas the other test line of the detector is connected to the ground (Figure 1(b)). The plant is placed inside an insulated flower pot. The sensor signal is tested in a shielded room to eliminate the interference of other signals on the test.

### 4.3. Fabrication of the Wireless Monitoring System

A Ti sheet is inserted into the soil of the green plant with a copper lead connected to the signal amplifier, whereas the other test line of the amplifier is connected to the ground. The amplified voltage is directly connected to a delay power switch. The delay power switch is connected to the wireless signal transmitter. The amplified voltage is used to trigger the wireless monitoring system. When the voltage signal of the amplifier reaches the prewarning value, the delay switch will be triggered to turn on the switch, and then the wireless transmitter will be powered on and transmit a wireless prewarning signal. When the wireless receiver receives the warning signal, it will send out an audible and visual alarm signal to warn of danger. Meanwhile, the wireless receiver is connected to the Internet, thus enabling the warning information to be uploaded to the cloud. The cloud can synchronize early warning information to mobile phones in real time. In this way, we realize a wireless early warning signal through a plant sensor and a wireless transmission system, which could also synchronize early warning notification to mobile phones.

### 4.4. Characterization

The short-circuit current was measured by a SR570 Low-Noise Current Amplifier (Stanford Research Systems, USA). The output voltage was tested through the NI-9215 (National Instruments) under a load resistance of 100 M*Ω*.

### 4.5. Theoretical Modelling

A theoretical model was developed in order to predict the amount of charge and current intensity produced during the triboelectric process (Supporting Information (available here)). Quantum mechanical calculations were performed on PTFE and NY, according to the protocol described in the Supporting Information. The computational results were plugged into the model.

## Data Availability

The experimental data used to support the findings of this study are available from the corresponding authors upon request.

## Supplementary Materials

Supplementary 1 Supporting information: additional figures and theoretical modelling for the manuscript.

Supplementary 2 Video S1: wireless monitoring sensor based on a plant sensor.

Supplementary 3 Video S2: real-time signal when stepping in front of a plant-based sensor.
