# Supplementary material for: Concealed Wireless Warning Sensor Based on Triboelectrification and Human-Plant Interactive Induction
Source: Research (Wash D C). 2021 Apr 29;2021:9870936. doi: 10.34133/2021/9870936 (PMC8106444; doi:10.34133/2021/9870936)
Supplement: Supplementary 1 — Supporting information: additional figures and theoretical modelling for the manuscript. [file 9870936.f1.docx]

*Supporting Information*

**Concealed Wireless Warning Sensor based on Triboelectrification and Human–Plant Interactive Induction**

**Concealed Wireless Warning Sensor based on Green Plant**

Yange Feng^1, 2, †^, Enrico Benassi,^1, 3, 4, †^ Liqiang Zhang^1^, Xiaojuan Li^1^, Daoai Wang,^1, 2,*^ Feng Zhou^1,*^, Weimin Liu^1,*^.

^1^ State Key Laboratory of Solid Lubrication, Lanzhou Institute of Chemical Physics, Chinese Academy of Sciences, Lanzhou 730000, China.

^2^ Qingdao Center of Resource Chemistry and New Materials, Qingdao 266100, China.

^3^ Public Technical Service Center, Lanzhou Institute of Chemical Physics, Chinese Academy of Sciences, Lanzhou 730000, China.

^4^ Novosibirsk State University, 1, Pirogova str., Novosibirsk, 630090, Russia.

* Correspondence should be addressed to D. Wang; wangda@licp.cas.cn, F. Zhou; zhouf@licp.cas.cn and W. Liu; wmliu@licp.cas.cn

^†^ The authors contribute equally to this work.

| **Figure S1** Detected current with Epipremnum aureum and Pachira macrocarpa. | p. SI-3 |
| --- | --- |
| **Figure S2**Current value measured at a distance interval of 2cm. | p. SI-3 |
| **Figure S3** (a) Detected current when stepping with different materials; (b) detected current when shoe soles in different humidity environment; (c) detected current when swing arms and stepping. | p. SI-4 |
| **Figure S4** (a) Photograph of the three different plants; (b) detected current when the three plants act as signal sensors. | p. SI-5 |
| **Figure S5** Detected current under different burden for two individuals, (a) A and (b) B; detected current in case of odd loads for (c) A and (d) B. | p. SI-6 |
| **Figure S6** Triboelectric signals generated by PTFE and NY films; current generated by silicon rubber with different size; and current generated by silicon rubber under different contact pressure. | p. SI-7 |
| **Theoretical Model and Quantum Chemical Calculations**  **1 Generation of Charge Distribution** | p. SI-8 |
| **2 Features of Charge Distribution** | p. SI-11 |
| **3 Separation of the Layers** | p. SI-15 |
| **4 Numerical Evaluation of the Model** | p. SI-19 |
| **5 Computational Details** | p. SI-20 |
| **References** | p. SI-21 |


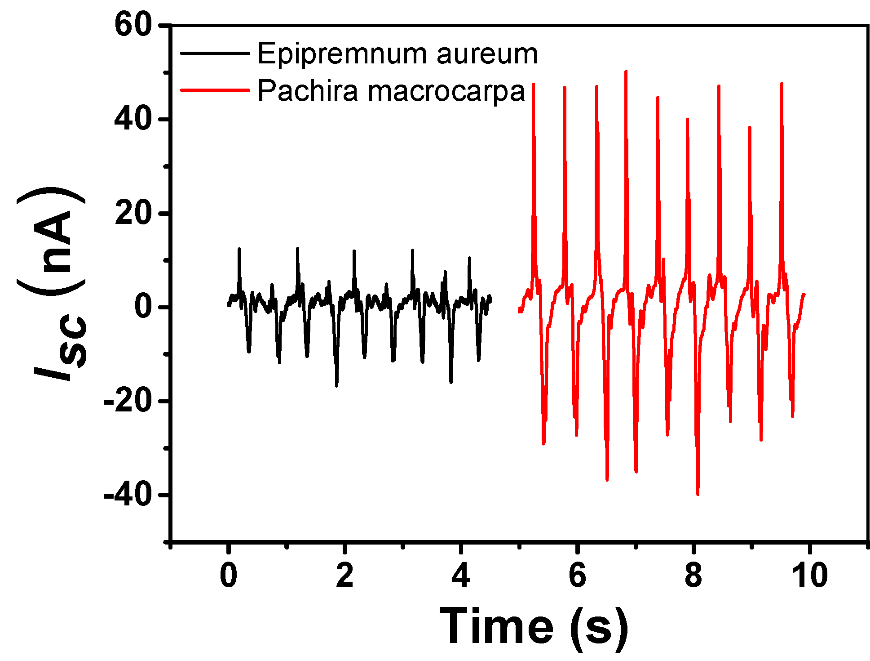


**Figure S1** Detected current with Epipremnum aureum and Pachira macrocarpa


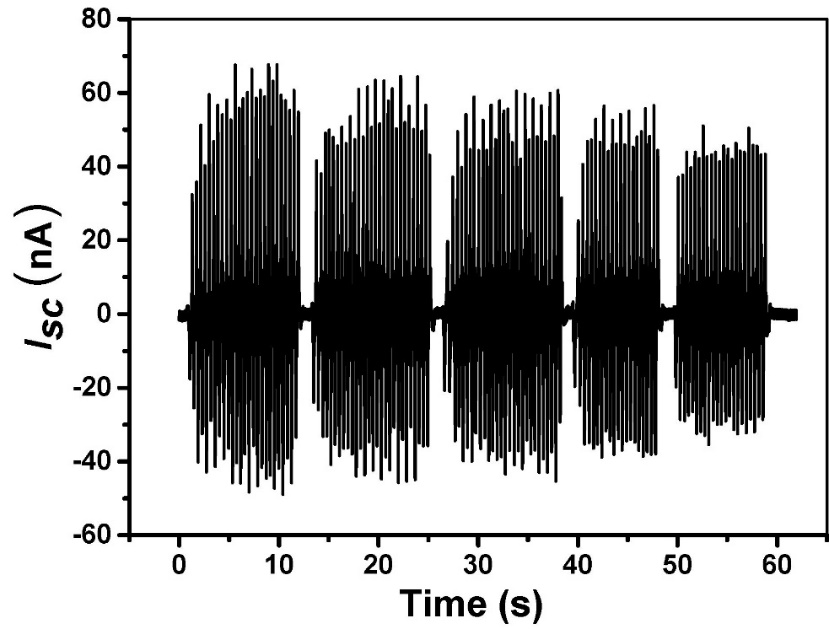


**Figure S2** Current value measured at a distance of 2cm.


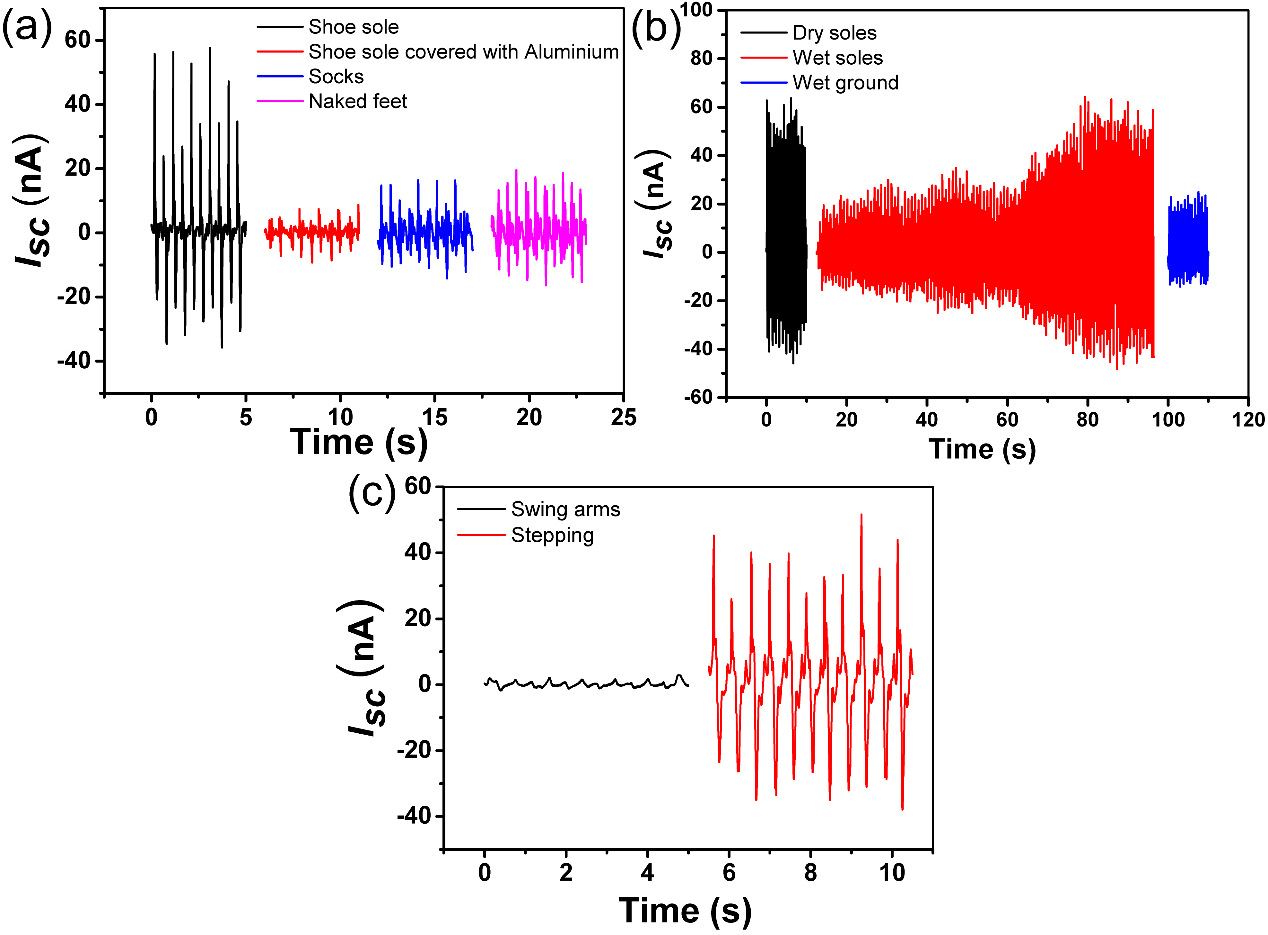


**Figure S3** (a) Detected current when stepping with different materials; (b) detected current when shoe soles in different humidity environment; (c) detected current when swing arms and stepping.


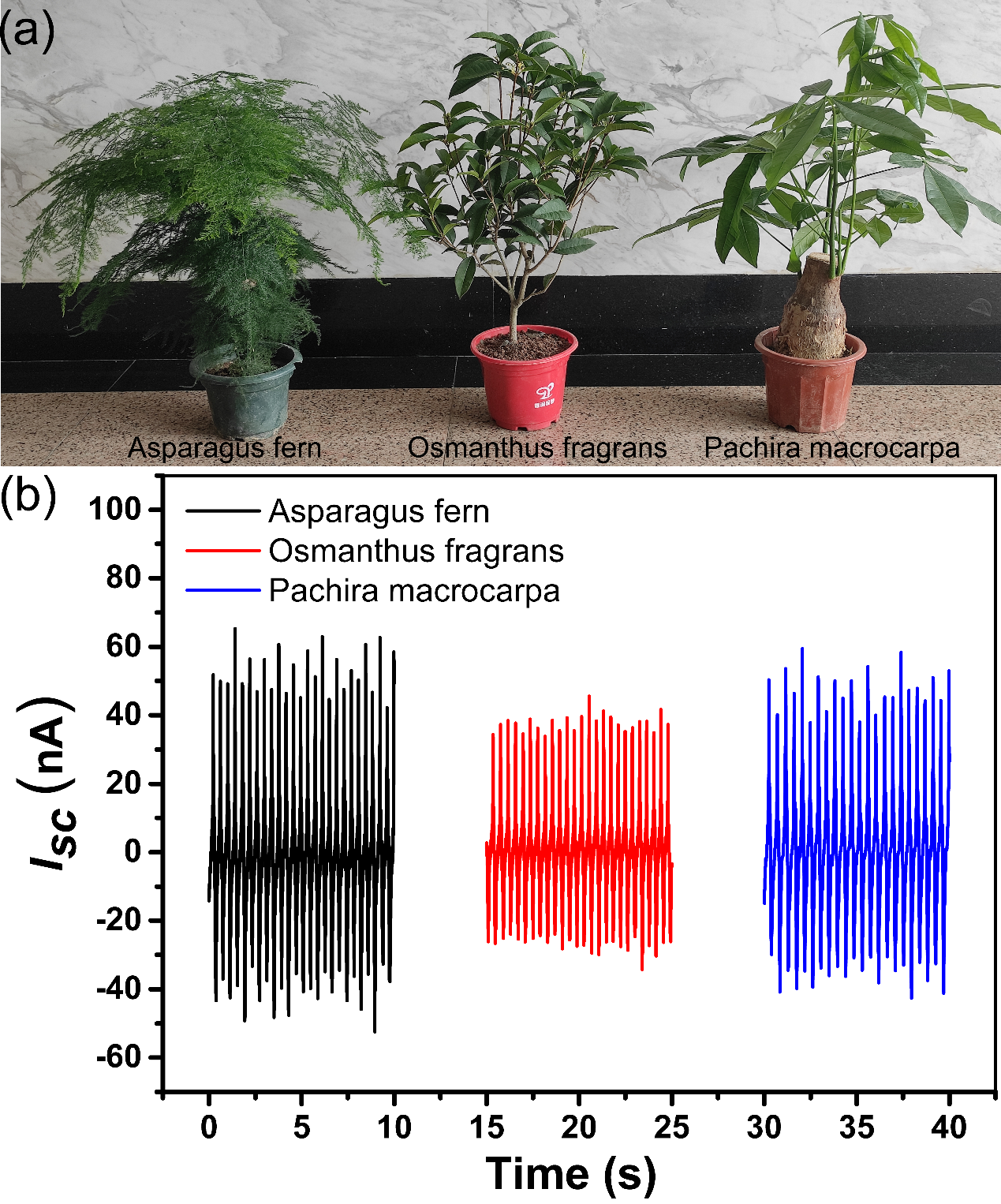


**Figure S4** (a) Photograph of the three different plants; (b) detected current when the three plants act as signal sensors.


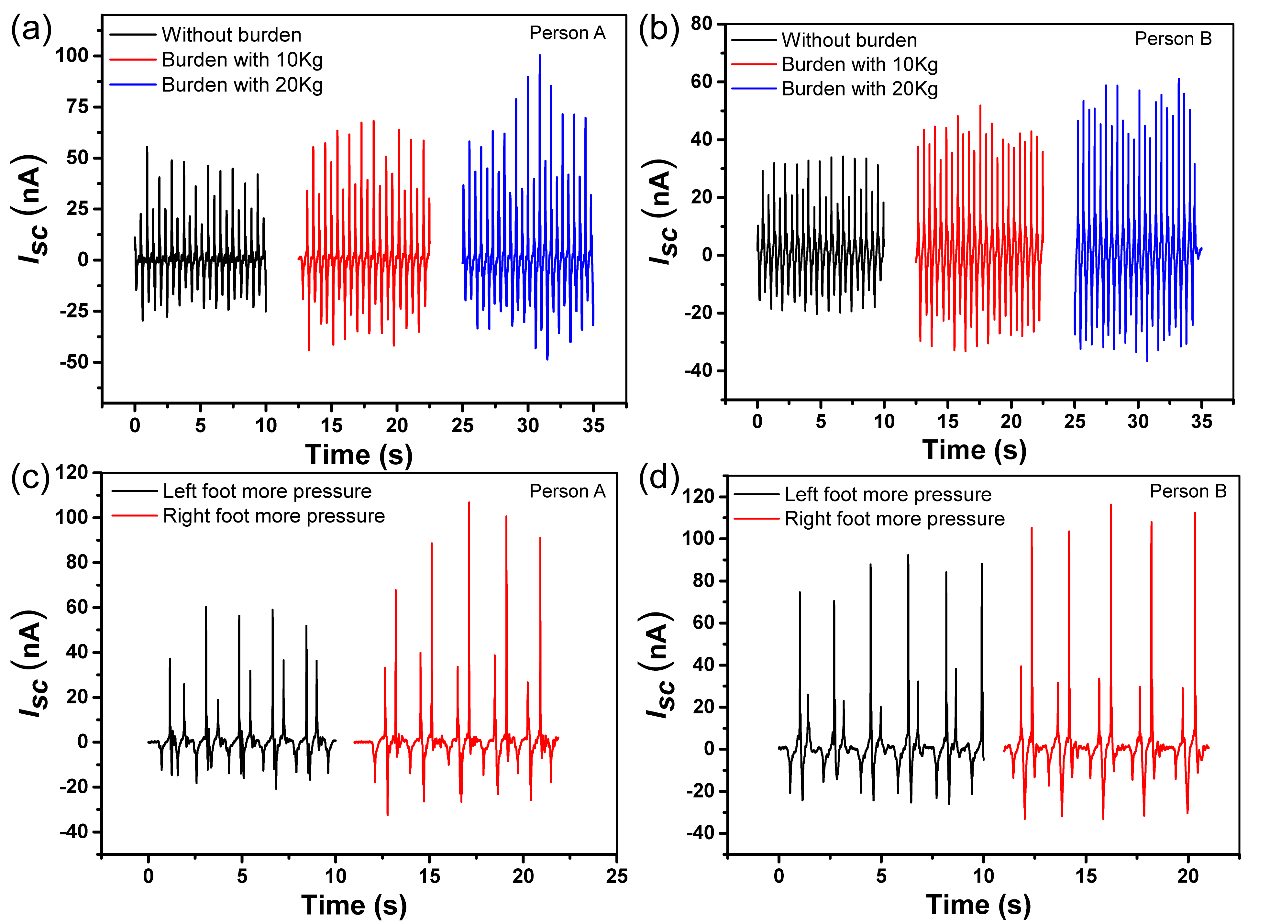


**Figure S5** Detected current under different burden for two individuals, (a) A and (b) B; detected current in case of odd loads for (c) A and (d) B.


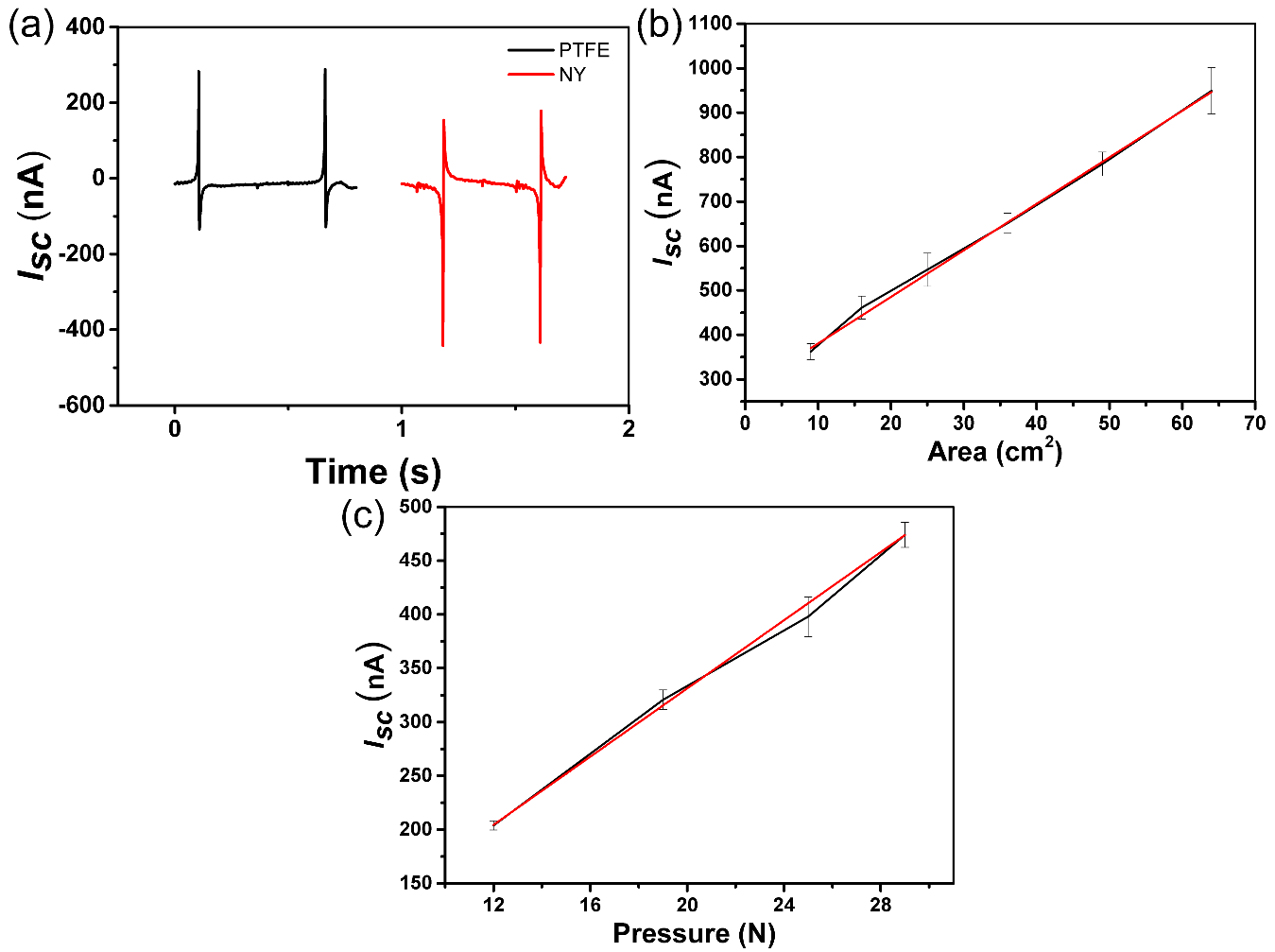


**Figure S6** (a) Triboelectric signals generated by PTFE and NY films; (b) current generated by silicon rubber with different size; (c) current generated by silicon rubber under different contact pressure.

**Video S1** Plant-based wireless early warning sensor system for danger early warning.

**Video S2** Plant-based wireless sensor for movement detection.

**Theoretical Model and Quantum Chemical Calculations**

**1 Generation of Charge Distribution**

When two materials with different dielectric properties are in contact to each other, at the interface that separates the two materials an electrochemical equilibrium is established. When the separation of the two materials occurs, a redistribution of the surface charges will take place. This is an out-of-equilibrium process and firstly involves the electronic polarisation, being the fastest to re-arrange (~ fs); atomic (~1/10 ps) and orientation polarization (10 ps) are involved in a second stage of the process. We therefore distinguish two types of polarisations: non-equilibrium, $\mathbf{P}_{neq}\left( \mathbf{r} \right)$, and equilibrium, $\mathbf{P}_{eq}\left( \mathbf{r} \right)$. The total polarisation is given by the sum of these two, *i.e.* $\mathbf{P}\left( \mathbf{r} \right)=\mathbf{P}_{neq}\left( \mathbf{r} \right)+\mathbf{P}_{eq}\left( \mathbf{r} \right)$. The value of the polarisations is supposed to vary on time in a different way; in particular, we may distinguish these two stages on the basis of the values assumed by the non-equilibrium and equilibrium polarisations. In the first stage, the final value of the non-equilibrium polarisation, namely $\mathbf{P}_{neq}^{0}\left( \mathbf{r} \right)$, is produced in a reversible way by formation of a charge distribution, whereas the equilibrium polarisation retains a certain value, namely $\mathbf{P}_{eq}^{0}\left( \mathbf{r} \right)$. In the second stage, the value of $\mathbf{P}_{neq}^{0}\left( \mathbf{r} \right)$ is held fixed but, due to the alteration of the charge distribution, the equilibrium polarisation passes from the value $\mathbf{P}_{eq}^{0}\left( \mathbf{r} \right)$ to the value $\mathbf{P}_{eq}\left( \mathbf{r} \right)$. The central quantity that has to be evaluated is the electrostatic potential:

$\psi\left( \mathbf{r}^{'} \right)=\int dS\frac{\sigma\left( \mathbf{r} \right)}{\left| \mathbf{r}-\mathbf{r}^{'} \right|}+\int dV\frac{\rho\left( \mathbf{r} \right)}{\left| \mathbf{r}-\mathbf{r}^{'} \right|}+\int dV \mathbf{P}\left( \mathbf{r} \right)\cdot\boldsymbol{\nabla}\frac{1}{\left| \mathbf{r}-\mathbf{r}^{'} \right|}$, (1.1)

where $\sigma\left( \mathbf{r} \right)$ and $\rho\left( \mathbf{r} \right)$ indicate the surface and volume charge density, respectively.

In the first stage, the initial values of charge densities ($\sigma^{0}\left( \mathbf{r} \right)$ and $\rho^{0}\left( \mathbf{r} \right)$), non-equilibrium polarisation $\mathbf{P}_{neq}^{0}\left( \mathbf{r} \right)$, the electric potential $\psi^{0}\left( \mathbf{r} \right)$, and the electric field $\mathbf{E}^{0}\left( \mathbf{r} \right)=-\boldsymbol{\nabla}\psi^{0}\left( \mathbf{r} \right)$ change their values due to the charging process. If we indicate with $x$ the fraction of charge ($x\in\left[ 0,1 \right]$), then we can write:

$\sigma^{x}\left( \mathbf{r} \right)=x\sigma^{0}\left( \mathbf{r} \right)$, (1.2)

$\rho^{x}\left( \mathbf{r} \right)=x\rho^{0}\left( \mathbf{r} \right)$, (1.3)

$\psi^{x}\left( \mathbf{r} \right)=x\psi^{0}\left( \mathbf{r} \right)$, (1.4)

$\mathbf{E}^{x}\left( \mathbf{r} \right)=-\boldsymbol{\nabla}\psi^{x}\left( \mathbf{r} \right)$. (1.5)

By mean of the electric field, the polarisations may be evaluated:

$\mathbf{P}_{eq}^{x}\left( \mathbf{r} \right)=\alpha_{eq}\mathbf{E}^{x}\left( \mathbf{r} \right)$, (1.6)

$\mathbf{P}_{neq}^{x}\left( \mathbf{r} \right)=\alpha_{neq}\mathbf{E}^{x}\left( \mathbf{r} \right)$. (1.7)

From eq. (1.1), the electric potential, before and after the charging process, reads:

$\psi^{x}\left( \mathbf{r}^{'} \right)=\int dS\frac{x\sigma^{0}\left( \mathbf{r} \right)}{\left| \mathbf{r}-\mathbf{r}^{'} \right|}+\int dV\frac{x\rho^{0}\left( \mathbf{r} \right)}{\left| \mathbf{r}-\mathbf{r}^{'} \right|}-\int dV\left( \alpha_{eq}+\alpha_{neq} \right)\boldsymbol{\nabla}\psi^{x}\left( \mathbf{r} \right)\cdot\boldsymbol{\nabla}\frac{1}{\left| \mathbf{r}-\mathbf{r}^{'} \right|}$,

(1.8)

$\psi^{0}\left( \mathbf{r}^{'} \right)=\int dS\frac{\sigma^{0}\left( \mathbf{r} \right)}{\left| \mathbf{r}-\mathbf{r}^{'} \right|}+\int dV\frac{\rho^{0}\left( \mathbf{r} \right)}{\left| \mathbf{r}-\mathbf{r}^{'} \right|}-\int dV\left( \alpha_{eq}+\alpha_{neq} \right)\boldsymbol{\nabla}\psi^{0}\left( \mathbf{r} \right)\cdot\boldsymbol{\nabla}\frac{1}{\left| \mathbf{r}-\mathbf{r}^{'} \right|}$,

(1.9)

where $\alpha_{eq}$ and $\alpha_{neq}$ indicate the equilibrium and non-equilibrium polarisabilities. Hence we can evaluate the electric work (electrostatic free energy) required in the first stage as:

$W^{'}=\int_{x} dx\left[ \int_{S} dS\psi^{x}\left( \frac{\partial\sigma^{x}}{\partial x} \right)+\int_{V} dV\psi^{x}\left( \frac{\partial\rho^{x}}{\partial x} \right) \right]$ $=\frac{1}{2}\left[ \int dS\psi^{0}\sigma^{0}+\int dV\psi^{0}\rho^{0} \right]$. (1.10)

In the second stage, the initial values of charge densities are those obtained at the end of the previous stage, *i.e.* $\sigma^{0}\left( \mathbf{r} \right)$ and $\rho^{0}\left( \mathbf{r} \right)$, and they will change to their final values, $\sigma\left( \mathbf{r} \right)$ and $\rho\left( \mathbf{r} \right)$:

$\sigma^{x}\left( \mathbf{r} \right)=\sigma^{0}\left( \mathbf{r} \right)+x\left[ \sigma\left( \mathbf{r} \right)-\sigma^{0}\left( \mathbf{r} \right) \right]$, (1.11)

$\rho^{x}\left( \mathbf{r} \right)=\rho^{0}\left( \mathbf{r} \right)+x\left[ \rho\left( \mathbf{r} \right)-\rho^{0}\left( \mathbf{r} \right) \right]$. (1.12)

The electric potential reads:

$\psi^{x}\left( \mathbf{r}^{'} \right)=\int dS\frac{\sigma^{0}\left( \mathbf{r} \right)+x\left[ \sigma\left( \mathbf{r} \right)-\sigma^{0}\left( \mathbf{r} \right) \right]}{\left| \mathbf{r}-\mathbf{r}^{'} \right|}+\int dV\frac{\rho^{0}\left( \mathbf{r} \right)+x\left[ \rho\left( \mathbf{r} \right)-\rho^{0}\left( \mathbf{r} \right) \right]}{\left| \mathbf{r}-\mathbf{r}^{'} \right|}+ +\int dV\left[ \mathbf{P}_{neq}^{0}\left( \mathbf{r} \right)-\alpha_{eq}\boldsymbol{\nabla}\psi^{x}\left( \mathbf{r} \right) \right]\cdot\boldsymbol{\nabla}\frac{1}{\left| \mathbf{r}-\mathbf{r}^{'} \right|}$, (1.13)

and hence for the entire process in stage II, the change in electric potential reads:

$\psi\left( \mathbf{r}^{'} \right)-\psi^{0}\left( \mathbf{r}^{'} \right)=\int dS\frac{\sigma\left( \mathbf{r} \right)-\sigma^{0}\left( \mathbf{r} \right)}{\left| \mathbf{r}-\mathbf{r}^{'} \right|}+\int dV\frac{\rho\left( \mathbf{r} \right)-\rho^{0}\left( \mathbf{r} \right)}{\left| \mathbf{r}-\mathbf{r}^{'} \right|}+ +\int dV\alpha_{eq}\boldsymbol{\nabla}\left[ \psi\left( \mathbf{r} \right)-\psi^{0}\left( \mathbf{r} \right) \right]\cdot\boldsymbol{\nabla}\frac{1}{\left| \mathbf{r}-\mathbf{r}^{'} \right|}$. (1.14)

We are therefore able to evaluate the electric work required in the second stage as:

$W^{''}=\frac{1}{2}\left[ \int dS\left( \psi+\psi^{0} \right)\left( \sigma-\sigma^{0} \right)+\int dV\left( \psi+\psi^{0} \right)\left( \rho-\rho^{0} \right) \right]$. (1.15)

So the electrostatic free energy change along with the whole process is:

$A=W^{'}+W^{''} =\frac{1}{2}\left[ \int dS\left( \psi\sigma+\psi^{0}\sigma-\psi\sigma^{0} \right)+\int dV\left( \psi\rho+\psi^{0}\rho-\psi\rho^{0} \right) \right]$. (1.16)

This expression is not very practical; we need to relate the free energy to quantities easier to control and measure. It is rather elementary that the electric field generated by a certain charge distribution of “*j*” type may be written as:

$\mathbf{E}_{cd}^{j}\left( \mathbf{r} \right)=-\boldsymbol{\nabla}\left\{ \int dS^{'}\frac{\sigma^{j}\left( \mathbf{r}^{'} \right)}{\left| \mathbf{r}-\mathbf{r}^{'} \right|}+\int dV^{'}\frac{\rho^{j}\left( \mathbf{r}^{'} \right)}{\left| \mathbf{r}-\mathbf{r}^{'} \right|} \right\}$. (1.17)

Hence, eq. (1.16) becomes:

$A=\frac{1}{2}\int dV\left\{ \frac{E_{cd}^{2}}{4\pi}-\left[ \mathbf{P}\cdot\left( \mathbf{E}_{cd}-\mathbf{E}_{cd}^{0} \right)+\mathbf{P}^{0}\cdot\mathbf{E}_{\mathrm{cd}} \right] \right\}$. (1.18)

Furthermore, since $\mathbf{P}^{0}=\mathbf{P}_{neq}^{0}+\alpha_{eq}\mathbf{E}^{0}$ and $\mathbf{P}=\mathbf{P}_{neq}^{0}+\alpha_{eq}\mathbf{E}$, and it is straightforward to prove that:

$\int dV\left[ \mathbf{P}^{0}\cdot\left( \mathbf{E}-\mathbf{E}_{cd} \right) \right]=\int dV\left[ \mathbf{P}\cdot\left( \mathbf{E}^{0}-\mathbf{E}_{cd}^{0} \right) \right]$, (1.19)

then after some rearrangement:

$A=\frac{1}{2}\int dV\left\{ \frac{E_{cd}^{2}}{4\pi}-\left[ \mathbf{P}\cdot\mathbf{E}_{cd}-\mathbf{P}_{neq}\cdot\tilde{\mathbf{E}} \right] \right\}$, (1.20)

where $\tilde{\mathbf{E}}\equiv\left( \mathbf{P}_{neq}/\alpha_{neq} \right)-\mathbf{E}$.

If we indicate $\mathbf{P}\left( \mathbf{r} \right)\equiv\mathbf{P}_{neq}\left( \mathbf{r} \right)-\alpha_{eq}\boldsymbol{\nabla}\psi\left( \mathbf{r} \right)$, from the equation field,

$\psi\left( \mathbf{r}^{'} \right)=\int dS\frac{\sigma\left( \mathbf{r} \right)+\mathbf{P}\left( \mathbf{r} \right)\cdot\hat{\mathbf{n}}}{\left| \mathbf{r}-\mathbf{r}^{'} \right|}+\int dV\frac{\rho\left( \mathbf{r} \right)-\boldsymbol{\nabla}\cdot\mathbf{P}\left( \mathbf{r} \right)}{\left| \mathbf{r}-\mathbf{r}^{'} \right|}$, (1.21)

wherein $\hat{\mathbf{n}}$ indicates the unit vector normal to the surface $S$, we are ready to derive:

$\left( 1+4\pi\alpha_{eq} \right)\Delta\psi\left( \mathbf{r}^{'} \right)=-4\pi\left( \rho-\boldsymbol{\nabla}\cdot\mathbf{P}_{neq} \right)$. (1.22)

Let us now consider the two media 1 and 2. The field equation may be specified as:

$\left( \frac{\partial}{\partial n_{1}}+\frac{\partial}{\partial n_{2}} \right)\psi=4\pi\left( \sigma+P_{n1}+P_{n2} \right)$, (1.23)

wherein $n_{j}\left( j=1,2 \right)$ are the normal vectors to the surface of separation, and:

$P_{nj}=P_{eq,nj}+P_{neq,nj} =-\alpha_{eq,j}\frac{\partial\psi}{\partial n_{j}}+P_{neq,nj}$, (1.24)

and, hence:

$\sum_{j} \left( 1+4\pi\alpha_{eq,j} \right)\frac{\partial\psi}{\partial n_{j}}=4\pi\left( \sigma+\sum_{j} P_{neq,nj} \right)$. (1.25)

**2 Features of Charge Distribution**

In the previous Paragraph we described the process of formation of the charge distribution and showed how to compute it. In the followings, we are interested in describing the features of the newly generated charge distribution at the interface. This model will be developed within the framework of the Classical Electrodynamic theory. From this perspective, the system may be modelled as a collection of *N* point charges $\left\{ q_{k}\left( \mathbf{r}_{k} \right) \right\}_{k=1,...N}$, located in a region of three dimensional Cartesian space. The region is supposed to have the characteristics of a continuum medium, with dielectric constant $\varepsilon_{1}$. This charge distribution is responsible of the generation of an electrostatic potential. We can identify the position of the *k*th charge with respect to a prefixed Cartesian system of coordinates by mean of a vector, $\mathbf{r}_{k}\equiv\left( \begin{matrix} x_{k} & y_{k} & z_{k} \end{matrix} \right)$, defining the position of the charge with respect to the origin of the Cartesian system of coordinates. The set of electric charges can be now supposed to be bounded within a closed geometrical surface $\Sigma$, which separate medium 1 from from the bulk of the surrounding space (medium 2), being this considered as a homogeneous, isotropic, continuum, dielectric medium, characterised by a dielectric constant, $\varepsilon_{2}$. In order to compute the Helmholtz free energy of polarisation of an arbitrary distribution of charges, we have to solve the Laplace equation (LE) or the Poisson-Boltzmann equation (PBE) for the electrostatic potential, $\psi$. The respective general solution will read:

$\psi_{\text{LE}}\left( \mathbf{r} \right)=\sum_{k} \frac{B_{k}}{\left| \mathbf{r}-\mathbf{r}_{k} \right|}$, (2.1)

$\psi_{\text{PBE}}\left( \mathbf{r} \right)=\sum_{k} \left( \frac{A_{k}}{\left| \mathbf{r}-\mathbf{r}_{k} \right|}e^{-\left| \mathbf{r}-\mathbf{r}_{k} \right|/\lambda}+\frac{B_{k}}{\left| \mathbf{r}-\mathbf{r}_{k} \right|}e^{\left| \mathbf{r}-\mathbf{r}_{k} \right|/\lambda} \right)$, (2.2)

respectively, where the sums run over all charges, and the constants $A_{k}$ and $B_{k}$ are obtained when appropriate boundary conditions are imposed. The boundary conditions needed to solve the problem are: (1) continuity of the electric potential across the boundary surface $\Sigma$, and (2) continuity of the normal component of the dielectric displacement vector across the boundary surface $\Sigma$. Once solved LE and/or PBE for the bounded problem, we are able to determine the potentials within medium 1, $\psi_{1}$, and within medium 2, $\psi_{2}$. The charges within 1 induce a polarisation in 2, giving rise to a reaction potential, $\psi_{R}\left( \mathbf{r} \right)$, which acts back on the charge distribution in 1. Once determined the analytical expression for $\psi_{R}\left( \mathbf{r} \right)$, the Helmholtz free energy of this interaction is just the difference between the reversible work of assembling the charge distribution in 1 in the presence of 2, and simply reads:

$A_{el}=\frac{1}{2}\sum_{k} q_{k}\left( \mathbf{r}_{k} \right)\psi_{R}\left( \mathbf{r}_{k} \right)$. (2.3)

According to the model proposed in Paragraph 1, the generated distribution of charges will reach the thermal equilibrium. The nonlinear homogeneous PBE may be conveniently written in polar coordinates:

$\left( \frac{\partial^{2}}{\partial\rho^{2}}+\frac{1}{\rho}\frac{\partial}{\partial\rho}+\frac{1}{\rho^{2}}\frac{\partial^{2}}{\partial\phi^{2}} \right)\psi\left( \mathbf{r} \right)+\frac{4\pi qnN}{\varepsilon}e^{-\frac{q\psi\left( \mathbf{r} \right)}{kT}} =0$. (2.4)

where $n\equiv\int_{\Omega} dVe^{-q\psi\left( \mathbf{r} \right)/kT}$ indicates the normalization factor, wherein the integration is over the volume under consideration, $\Omega$, $N$ indicates the number of free ions with charge $q$ per unit axial length. In order to make it easier to solve, we may introduce auxiliary variables:

$\lambda_{D}\equiv q^{2}/kT$, $f_{D}\equiv\sqrt{\frac{4\pi nN}{\varepsilon}\lambda_{D}}$,

$\tilde{\rho}\equiv\rho f_{D}$, $\xi\equiv\ln\tilde{\rho}$, $\tilde{\psi}\left( \mathbf{r} \right)\equiv-\frac{q\psi\left( \mathbf{r} \right)}{kT}+2\xi$. (2.5)

Now eq. (2.4) reads:

$\frac{\partial^{2}\tilde{\psi}\left( \mathbf{r} \right)}{\partial\xi^{2}}+\frac{\partial^{2}\tilde{\psi}\left( \mathbf{r} \right)}{\partial\phi^{2}}=e^{\tilde{\psi}\left( \mathbf{r} \right)}$. (2.6)

By using Bäcklund’s transformation, the solutions of eq. (2.6) may be expressed through arbitrary harmonic functions, $Y\left( \xi,\phi\right)$:

$\tilde{\psi}\left( \mathbf{r} \right)=\ln\left\{ 2\frac{\left( \frac{\partial Y\left( \xi,\phi\right)}{\partial\xi} \right)^{2}+\left( \frac{\partial Y\left( \xi,\phi\right)}{\partial\phi} \right)^{2}}{\left[ Y\left( \xi,\phi\right) \right]^{2}} \right\}$, (2.7)

and thus for the electric potential solution of the PBE we get:

$\psi\left( \mathbf{r} \right)=-\frac{kT}{q}\ln\left[ 2\frac{\left( \tilde{\rho}\frac{\partial Y}{\partial\tilde{\rho}} \right)^{2}+\left( \frac{\partial Y}{\partial\phi} \right)^{2}}{\tilde{\rho}^{2}Y^{2}} \right]$. (2.8)

In order to solve analytically and in closed form the problem, we need to assume some special symmetry of the problem. We believe it might be appropriate to consider the shape of medium 1 at the interface as a longitudinal portion of cylinder of radius *a* and length *L*. In order to keep the problem rather general, so to also account for other charges generated by induction or simply mirror charges, we consider 1 as surrounded by another region, still cylindrical, of radius *b*, *i.e.* a co-axial, homogeneous, isotropic, continuum, dielectric shell, including a certain proper charge distribution, characterised by a dielectric constant, $\varepsilon_{shell}$. In cylindrical coordinates, the position of charges is described by the vector $\mathbf{r}_{k}\equiv\left( \begin{matrix} \rho_{k} & \theta_{k} & z_{k} \end{matrix} \right)$. After some elementary calculus, we find that for the cylindrical cavity eq. (2.3) reads:

$A_{el}=\frac{1}{\varepsilon_{1}L}\sum_{kl} q_{k}q_{l}\left( \psi_{0}+\psi_{1}+\psi_{2}+\psi_{4} \right)$, (2.9)

where:

$\psi_{0}\equiv\left( \frac{1}{\varepsilon_{a}}-1 \right)\left[ \frac{1}{\varepsilon_{b}}\frac{\mathcal{K}_{0}\left( b/\lambda\right)}{\left( b/\lambda\right)\mathcal{K}_{1}\left( b/\lambda\right)}+\ln\frac{b}{a} \right]+\left[ \frac{1}{\varepsilon_{b}}\frac{\mathcal{K}_{0}\left( b/\lambda\right)}{\left( \kappa b \right)\mathcal{K}_{1}\left( b/\lambda\right)}+\ln b \right]$, (2.10a)

$\psi_{1}\equiv\sum_{m=1}^{\infty} \frac{\cos\left[ m\left( \theta_{l}-\theta_{k} \right) \right]}{m}\rho_{l}^{m}\rho_{k}^{m}\left[ \left( \frac{1}{\varepsilon_{a}}-1 \right)\left( \frac{1}{a^{2m}}+\frac{\beta_{m}}{b^{2m}} \right)+\frac{\beta_{m}}{b^{2m}} \right]$, (2.10b)

$\psi_{2}\equiv2\sum_{n=1}^{\infty} \cos\left[ \lambda_{n}\left( z_{l}-z_{k} \right) \right]\mathcal{I}_{0}\left( \lambda_{n}\rho_{l} \right)\mathcal{I}_{0}\left( \lambda_{n}\rho_{k} \right)\left[ \left( 1-\varepsilon_{a}^{''} \right)\frac{\mathcal{K}_{1}\left( \lambda_{n}a \right)}{\mathcal{I}_{1}\left( \lambda_{n}a \right)}+\varepsilon_{a}^{''}\Psi_{n}^{0} \right]$, (2.10c)

$\psi_{4}\equiv4\sum_{m=1}^{\infty} \cos\left[ m\left( \theta_{l}-\theta_{k} \right) \right]\times\sum_{n=1}^{\infty} \left\{ \cos\left[ \lambda_{n}\left( z_{l}-z_{k} \right) \right]\mathcal{I}_{m}\left( \lambda_{n}\rho_{l} \right)\mathcal{I}_{m}\left( \lambda_{n}\rho_{k} \right)\left[ -\left( 1-\varepsilon_{a}^{'} \right)\frac{\mathcal{K}_{1}^{'}\left( \lambda_{n}a \right)}{\mathcal{I}_{1}^{'}\left( \lambda_{n}a \right)}+\varepsilon^{'}\Psi_{n}^{m} \right] \right\}$. (2.10d)

In these equations, the functions $\mathcal{K}_{m}\left( \xi\right)$ and $\mathcal{I}_{m}\left( \xi\right)$ are the modified Bessel functions of *m*th order, and $\mathcal{K}_{m}^{'}\left( \xi\right)$ and $\mathcal{I}_{m}^{'}\left( \xi\right)$ their respective derivatives, computed as:

$\mathcal{K}_{m}^{'}\left( \xi\right)=\frac{m}{\xi}\mathcal{K}_{m}\left( \xi\right)-\mathcal{K}_{m+1}\left( \xi\right)=-\left[ \frac{m}{\xi}\mathcal{K}_{m}\left( \xi\right)+\mathcal{K}_{m-1}\left( \xi\right) \right]$, (2.11a)

$\mathcal{I}_{m}^{'}\left( \xi\right)=\frac{m}{\xi}\mathcal{I}_{m}\left( \xi\right)+\mathcal{I}_{m+1}\left( \xi\right)=-\left[ \frac{m}{\xi}\mathcal{I}_{m}\left( \xi\right)-\mathcal{I}_{m-1}\left( \xi\right) \right]$. (2.11b)

In equations (2.10b) to (2.10d), the convergence of series needs to be defined by a threshold value. The meaning of the other auxiliary variables and functions is explained hereafter.

$\lambda_{n}\equiv2\pi n/L$, (2.12a)

$\mu_{n}\equiv\sqrt{\lambda_{n}^{2}+\left( 1/\lambda\right)^{2}}$, (2.12b)

$\varepsilon_{a}\equiv\varepsilon_{shell}/\varepsilon_{1}$, (2.12c)

$\varepsilon_{b}\equiv\varepsilon_{2}/\varepsilon_{shell}$, (2.12d)

$\beta_{m}\equiv\frac{m\mathcal{K}_{m}\left( b/\lambda\right)+\varepsilon_{b}\left( b/\lambda\right)\mathcal{K}_{m}^{'}\left( b/\lambda\right)}{m\mathcal{K}_{m}\left( b/\lambda\right)-\varepsilon_{b}\left( b/\lambda\right)\mathcal{K}_{m}^{'}\left( b/\lambda\right)}$, (2.12e)

$\Psi_{n}^{0}\equiv\frac{-\varepsilon_{b}\mu_{n}\mathcal{K}_{1}\left( \mu_{n}b \right)\mathcal{K}_{0}\left( \lambda_{n}b \right)+\lambda_{n}\mathcal{K}_{0}\left( \mu_{n}b \right)\mathcal{K}_{1}\left( \lambda_{n}b \right)}{\varepsilon_{b}\mu_{n}\mathcal{K}_{1}\left( \mu_{n}b \right)\mathcal{I}_{0}\left( \lambda_{n}b \right)+\lambda_{n}\mathcal{K}_{0}\left( \mu_{n}b \right)\mathcal{I}_{1}\left( \lambda_{n}b \right)}$, (2.12f)

$\Psi_{n}^{m}\equiv\frac{\varepsilon_{b}\mu_{n}\mathcal{K}_{m}^{'}\left( \mu_{n}b \right)\mathcal{K}_{m}\left( \lambda_{n}b \right)-\lambda_{n}\mathcal{K}_{m}\left( \mu_{n}b \right)\mathcal{K}_{m}^{'}\left( \lambda_{n}b \right)}{-\varepsilon_{b}\mu_{n}\mathcal{K}_{m}^{'}\left( \mu_{n}b \right)\mathcal{I}_{m}\left( \lambda_{n}b \right)+\lambda_{n}\mathcal{K}_{m}\left( \mu_{n}b \right)\mathcal{I}_{m}^{'}\left( \lambda_{n}b \right)}$, (2.12g)

$\varepsilon_{a}^{'}\equiv\left\{ \frac{\lambda_{n}a}{\varepsilon_{a}}\left[ \begin{aligned} &\left( 1-\varepsilon_{a} \right)\Psi_{n}^{m}\mathcal{I}_{m}\left( \lambda_{n}a \right)\mathcal{I}_{m}^{'}\left( \lambda_{n}a \right)+\mathcal{K}_{m}\left( \lambda_{n}a \right)\mathcal{I}_{m}^{'}\left( \lambda_{n}a \right)+ \\ &-\varepsilon_{a}\mathcal{K}_{m}^{'}\left( \lambda_{n}a \right)\mathcal{I}_{m}\left( \lambda_{n}a \right) \end{aligned} \right] \right\}^{-1}$, (2.12h)

$\varepsilon_{a}^{''}\equiv\left\{ \frac{\lambda_{n}a}{\varepsilon_{a}}\left[ \begin{aligned} &\left( 1-\varepsilon_{a} \right)\Psi_{n}^{0}\mathcal{I}_{0}\left( \lambda_{n}a \right)\mathcal{I}_{1}\left( \lambda_{n}a \right)+\mathcal{K}_{0}\left( \lambda_{n}a \right)\mathcal{I}_{1}\left( \lambda_{n}a \right)+ \\ &+\varepsilon_{a}\mathcal{K}_{1}\left( \lambda_{n}a \right)\mathcal{I}_{1}\left( \lambda_{n}a \right) \end{aligned} \right] \right\}^{-1}$. (2.12i)

**3 Separation of the Layers**

The experimental setup, as described in the main text, may be modelled as a “sandwich” constituted of layers of different materials (and/or vacuum). In particular we consider two layers of a metal material M’ and a dielectric material D (sample) in contact to each other, separated by another layer of the same metal material M” by an empty space V. Each layer has a parallelepiped shape; D has fixed thickness $\delta$, and V has variable thickness, $z\left( t \right)$. The thickness $z$ varies in time from an initial value 0 ($t_{0}\equiv0$; when M’D is in direct contact with M”) to a final value $z_{max}$ ($t_{max}$; when M’D is separated by the other M”). In our experiments, $z$ varies in time with constant velocity, $\dot{z}\left( t \right)\equiv v=v_{0}$, and hence

$z\left( t \right)=v_{0}t,t\in\left[ 0,t_{max} \right]$. (3.1)

At this point, we shall make a distinction, *viz.* when D is constituted (a) by one material only (*viz.*, N and F samples) or (b) by a series of “strips”, each of them constituted by materials with different dielectric properties (*viz.*, 2-, 4-, 6-, and 8ED samples).

*Case (a)*

In this case the modelling is rather simple, consisting of two capacitors in series, one filled by D, one evacuated. Border effects can be neglected. The potential is the sum of the two potentials, *i.e.*:

$\psi=\psi_{D}+\psi_{V}$. (3.2)

Since the electric fields can be considered as uniform,

$E_{D}=-\frac{\tilde{\sigma}}{\varepsilon_{0}\varepsilon_{D}}$, (3.3a)

$E_{V}=-\frac{\tilde{\sigma}+\sigma}{\varepsilon_{0}}$, (3.3b)

being $\tilde{\sigma}\equiv q_{0}/\Sigma$, we can write:

$\psi=-\frac{\tilde{\sigma}}{\varepsilon_{0}}\left[ \tilde{\delta}+z\left( t \right) \right]+\frac{\sigma}{\varepsilon_{0}}z\left( t \right)$, (3.4)

where $\tilde{\delta}\equiv\delta/\varepsilon_{D}$. We can calculate the amount of charge as a function of time by the first Ohm law:

$q\left( t \right)=q_{0}\left\{ 1-\eta\left( t \right)\left[ 1+\frac{\tilde{\delta}}{R\Sigma\varepsilon_{0}}\int_{0}^{t} dt^{'}\eta\left( t^{'} \right) \right] \right\}$, (3.5)

where:

$\eta\left( t \right)\equiv\exp\left[ -\frac{1}{R\Sigma\varepsilon_{0}}\left( \tilde{\delta}t+\int_{0}^{t} d\tau z\left( \tau\right) \right) \right]$. (3.6)

From eq. (3.5), the current intensity, $i\left( t \right)\equiv\dot{q}\left( t \right)$, and the potential, $\psi\left( t \right)\equiv Ri\left( t \right)$, can be readily computed. By plugging eq. (3.1) into eq. (3.5), we get:

$q\left( t \right)=q_{0}\left\{ 1-e^{-\alpha^{2}v_{0}\left( 2\tilde{\delta}+v_{0}t \right)t} \left[ 1+\sqrt{\pi}\alpha\tilde{\delta}e^{\alpha^{2}\tilde{\delta}^{2}} \left( \mathrm{erf}\left[ \alpha\left( \tilde{\delta}+v_{0}t \right) \right]-\mathrm{erf}\left[ \alpha\tilde{\delta} \right] \right) \right] \right\}$,

(3.7)

where $\alpha\equiv\left[ 2v_{0}R\Sigma\varepsilon_{0} \right]^{-1/2}$.

*Case (b)*

With respect to case (a), this scenario is complicated by two factors. Firstly, D has to be considered as constituted by capacitors in parallel. Secondly, border effects cannot be neglected. The first complication is easy to treat, by laws of elementary Electrodynamics. We just underline that if we consider D as made of two materials, namely A and B, of different dielectric constants, $\varepsilon_{A}$ and $\varepsilon_{B}$, then the two electric fields $\mathbf{E}_{A}$ and $\mathbf{E}_{B}$ will still have the same intensity; yet, since the dielectric are different, then the surface charge densities, $\sigma_{A}$ and $\sigma_{B}$, will be different. Moreover, in the interface separating A and B, a force of electrostatic nature will generate, having direction corresponding to the normal to the interface and intensity:

$\boldsymbol{F}=-\boldsymbol{\nabla}W=\frac{\left( \Delta\psi\right)^{2}\mathcal{l}\Delta\varepsilon}{2\delta}\hat{\mathbf{n}}$, (3.8)

where $\delta$ and $\mathcal{l}$ indicate the size of the (supposedly rectangular) interface between A and B, and $\Delta\varepsilon\equiv\left| \varepsilon_{A}-\varepsilon_{B} \right|$.

Now we want to evaluate the border effect. From a qualitative point of view, we may describe the phenomenon as follows. In proximity of the border of a capacitor the electric field cannot be considered as constant or uniform as in the central portion of the device. Force lines exceed the confinement of the parallelepiped which geometrically defines the capacitor. This implies an effect of polarisation on the material filling the neighbouring region. To depict a quantitative picture of the physics of the problem, we start writing the expression of the electrostatic potential in rectangular coordinates:

$\psi\left( x,y,z \right)=-\frac{1}{4\pi}\iint dx^{'}dy^{'}\psi\left( x^{'},y^{'},0 \right)\frac{\partial\mathcal{G}}{\partial n^{'}}$, (3.9)

where $n^{'}$ is the normal, and the derivative of the Green function $\mathcal{G}$ reads:

$\frac{\partial\mathcal{G}}{\partial n^{'}}\equiv\left( -\frac{\partial\mathcal{G}}{\partial z^{'}} \right)_{z^{'}=0}=\frac{-2z}{\left[ \left( x-x^{'} \right)^{2}+\left( y-y^{'} \right)^{2}+z^{2} \right]^{3/2}}$. (3.10)

If the two faces have potential $\left( \pm V/2 \right)$, then

$\psi\left( x,y,z \right)=\frac{V}{4\pi}z\iint\frac{dx^{'}dy^{'}}{\left[ \left( x-x^{'} \right)^{2}+\left( y-y^{'} \right)^{2}+z^{2} \right]^{3/2}}$. (3.11)

If we consider a ($\mathcal{l}\times w$) rectangular shape, by integrating over $dx^{'}$, we may compute the electrostatic potential and the electric field along the *z*-axis:

$\psi\left( 0,0,z \right)=\frac{V}{\pi}\arctan\left( \frac{\mathcal{l}w}{2z\sqrt{\mathcal{l}^{2}+w^{2}+4z^{2}}} \right)$, (3.12a)

$E_{z}\left( 0,0,z \right)=\frac{2\mathcal{l}wV\left( \mathcal{l}^{2}+w^{2}+8z^{2} \right)}{\pi\left( \mathcal{l}^{2}+4z^{2} \right)\left( w^{2}+4z^{2} \right)\sqrt{\mathcal{l}^{2}+w^{2}+4z^{2}}}$, (3.12b)

and on the (*x* = 0)-plane:

$\psi\left( 0,y,z \right)=\frac{V}{2\pi}\left\{ \arctan\left[ \frac{\mathcal{l}\left( w-2y \right)}{2z\sqrt{\mathcal{l}^{2}+\left( w-2y \right)^{2}+4z^{2}}} \right]+\arctan\left[ \frac{\mathcal{l}\left( w+2y \right)}{2z\sqrt{\mathcal{l}^{2}+\left( w+2y \right)^{2}+4z^{2}}} \right] \right\}$, (3.13a)

$E_{z}\left( 0,y,z \right)=\frac{\mathcal{l}V}{\pi\left( \mathcal{l}^{2}+4z^{2} \right)}\left\{ \frac{\left( w-2y \right)\left[ \mathcal{l}^{2}+\left( w-2y \right)^{2}+8z^{2} \right]}{\left[ \left( w-2y \right)^{2}+4z^{2} \right]\sqrt{\mathcal{l}^{2}+\left( w-2y \right)^{2}+4z^{2}}}+\frac{\left( w+2y \right)\left[ \mathcal{l}^{2}+\left( w+2y \right)^{2}+8z^{2} \right]}{\left[ \left( w+2y \right)^{2}+4z^{2} \right]\sqrt{\mathcal{l}^{2}+\left( w+2y \right)^{2}+4z^{2}}} \right\}$,(3.13b)

$E_{y}\left( 0,y,z \right)=\frac{2\mathcal{l}Vz}{\pi}\left\{ \frac{1}{\left[ \left( w-2y \right)^{2}+4z^{2} \right]\sqrt{\mathcal{l}^{2}+\left( w-2y \right)^{2}+4z^{2}}}-\frac{1}{\left[ \left( w+2y \right)^{2}+4z^{2} \right]\sqrt{\mathcal{l}^{2}+\left( w+2y \right)^{2}+4z^{2}}} \right\}$,(3.13c)

**4 Numerical Evaluation of the Model**

The described model was applied to calculate the surface charge density as a function of the composition of the system. The dielectric properties of materials were computed at Density Functional Theory (DFT) level (see below). Deviation from the linearity is due to the fact that this model does not account for some effects (mainly border, field penetration and back-polarisation effects), usually neglected, but that increase in importance when the dimensionalities thickness and depth are comparable.

Concerning the simulation of the dependence on time of the charge density when two different materials are placed in contact and then separated, the model was used to calculate the evolution of the percentage of surface charge density as a function of time. The mechanism of the phenomenon has been described by a model that accounts for two steps; each step has a different kinetics. In particular, we may formalise the time decay as follows:

$\sigma_{\%}\left( t \right)=\sigma_{\%}\left( \infty\right)+\sigma_{\%1}e^{-t/\tau_{1}} +\sigma_{\%2}e^{-t/\tau_{2}}$, (4.1)

where the two characteristic times, $\tau_{1}$ and $\tau_{2}$, refer to the two processes, and $\sigma_{\%1}$ and $\sigma_{\%2}$ describe the weight of each process. This description (with two exponential functions and not just one, wherein the weight of the two functions depends on the material segmentation, *N*) is also in agreement on what experimentally observed, since for bigger *N* a rapid decrease (a kind of “sudden jump”) in the very first instants and then a “less sudden” behaviour for longer times are shown. This could not be explained assuming one-exponential decay (that means a single process in the mechanism).

The off-set ($\sigma_{\%}\left( \infty\right)$) indicates the residual charge density at infinite time. This quantity does not linearly depend on *N*; this is reasonable because when *N* tends to infinity (a situation that we may chemically interpret as a “mixture” of the two materials), this quantity has to tend to a “saturation” value (namely, $\sigma_{\%}\left( \infty\right)$), and not to decrease linearly (to minus infinity). We can derive:

$\sigma_{\%}\left( \infty\right)=\sigma_{\%}^{\left( sat \right)}\left( \infty\right)+Ae^{-kN}$, (4.2)

**5 Computational Details**

In order to compute the dielectric properties of the materials, we performed quantum mechanical calculations on the monomers and small aggregates. The molecular geometries were fully optimised both in the gas phase at Density Functional Theory (DFT) level. The vibrational frequencies and thermochemicals were computed in harmonic approximation at *T* = 298.15 K and *p* = 1 atm, and no imaginary frequencies were found.

The calculations were performed by using M06-2X [1] DFT functionals in combination with 6-311++G** [2]. The static molecular polarizability was computed as suggested in ref. [3]. The dielectric constant and refractive index were computed by mean of the Mossotti-Clausius and Lorenz-Lorentz formulae, respectively, using the experimental values of density at 25 ^o^C.

Integration grid for the electronic density was set to 250 radial shells and 974 angular points. Convergence criteria of Self-Consistent Field were set to 10^-12^ for root mean square (RMS) change in density matrix and 10^-10^ for maximum change in density matrix. Convergence criteria for optimizations were set to 2 × 10^-6^ a.u. for maximum force, 1 × 10^-6^ a.u. for RMS force, 6 × 10^-6^ a.u. for maximum displacement and 4 × 10^-6^ a.u. for RMS displacement.

All calculations were performed using GAUSSIAN G09.D01 package [4].

**References**

[1] Y. Zhao, D. G. Truhlar, *Theor. Chem. Acc.* **2008**, *120*, 215-241.

[2] a) A. D. McLean, G. S. Chandler, *J. Chem. Phys.* **1980**, *72*, 5639-5648. b) K. Raghavachari, J. S. Binkley, R. Seeger, J. A. Pople, *J. Chem. Phys.* **1980**, *72*, 650-654. c) R. C. Binning Jr., L. A. Curtiss, *J. Comp. Chem.* **1990**, *11*, 1206-1216. d) M. P. McGrath, L. Radom, *J. Chem. Phys.* **1991**, *94*, 511-516. e) L. A. Curtiss, M. P. McGrath, J.-P. Blaudeau, N. E. Davis, R. C. Binning Jr., L. Radom, *J. Chem. Phys.* **1995**, *103*, 6104-6113.

[3] E. Benassi, F. Egidi, V. Barone, *J Phys Chem B* **2015**, *119*, 3155-3173.

[4] M. J. Frisch, G. W. Trucks, H. B. Schlegel, G. E. Scuseria, M. A. Robb, J. R. Cheeseman, G. Scalmani, V. Barone, B. Mennucci, G. A. Petersson, H. Nakatsuji, M. Caricato, X. Li, H. P. Hratchian, A. F. Izmaylov, J. Bloino, G. Zheng, J. L. Sonnenberg, M. Hada, M. Ehara, K. Toyota, R. Fukuda, J. Hasegawa, M. Ishida, T. Nakajima, Y. Honda, O. Kitao, H. Nakai, T. Vreven, J. A. Montgomery Jr., J. E. Peralta, F. Ogliaro, M. Bearpark, J. J. Heyd, E. Brothers, K. N. Kudin, V. N. Staroverov, R. Kobayashi, J. Normand, K. Raghavachari, A. Rendell, J. C. Burant, S. S. Iyengar, J. Tomasi J, M. Cossi, N. Rega, J. M. Millam, M. Klene, J. E. Knox, J. B. Cross, V. Bakken, C. Adamo, J. Jaramillo, R. Gomperts, R. E. Stratmann, O. Yazyev, A. J. Austin, R. Cammi, C. Pomelli, J. W. Ochterski, R. L. Martin, K. Morokuma, V. G. Zakrzewski, G. A. Voth, P. Salvador, J. J. Dannenberg, S. Dapprich, A. D. Daniels, O. Farkas, J. B. Foresman, J. V. Ortiz, J. Cioslowski, D. J. Fox. Gaussian09, revision D.01; Gaussian, Inc.: Wallingford, CT, **2009**.
